# The Top 100 Most Cited Journal Articles in Pediatric Neurosurgery

**DOI:** 10.7759/cureus.20694

**Published:** 2021-12-25

**Authors:** Viktoriya Grayson, Mitchell W Couldwell, Esther Dupepe, Joe Iwanaga, CJ Bui, Aaron S Dumont, R. Shane Tubbs

**Affiliations:** 1 Department of Neurosurgery, Tulane University School of Medicine, New Orleans, USA; 2 Department of Pediatric Neurosurgery, Ochsner Louisiana State University Health, New Orleans, USA; 3 Department of Neurology, Tulane University School of Medicine, New Orleans, USA; 4 Department of Neurosurgery, Ochsner Neuroscience Institute, Ochsner Health System, New Orleans, USA; 5 Anatomical Sciences, St. George's University, St. George's, GRD

**Keywords:** category, citation, journal, publication, pediatric neurosurgery

## Abstract

With the many papers published in the field of pediatric neurosurgery, it is often difficult to recognize those that have the most impact on future papers, i.e., citable papers. However, citation analysis allows one to better understand which papers are impacting the field the most. Therefore, the current study aimed to evaluate this literature. The Journal Citation Report database was searched for publications with the words “pediatric neurosurgery” or “pediatric neuro” in the title. Using the Web of Science Core Collection, the top 100 journal articles in pediatric neurosurgery from the selected journals were identified and citation analysis was used to identify the most impactful articles. A search was performed on Web of Science Core Collection by searching for each journal under “Publication Name” and using the Boolean “OR” function to separate fields. The results were ordered by the “Times Cited” category, which provided a list of all the articles from the eight journals appearing in the most cited order. The timeline used was from 1976 to 2021. The top 100 most cited articles were extracted from this list for analysis. The following variables were collected from each scientific article: publication journal, impact factor of journal, title, number of citations, year and month of publication, and type of article. Eight journals were identified on the basis of our search criteria and the articles were sorted by most cited; 1609 pediatric neurosurgery journal articles were screened to select the 100 most cited since 1976. This compilation could serve to help clinicians and researchers to familiarize themselves with the journal articles included in terms of study type, study field, journal of publication, and recurring authors.

## Introduction and background

There are many journal articles published on pediatric neurosurgery; a way to analyse and compare these publications is using citation analysis. The first scientific database for citation tracking was developed by the Institute for Scientific Information in 1962, later combined with the Social Sciences Citation Index in 1973 and the Arts and Humanities Citation Index in 1978. In 1997, this database was presented online under the name Web of Science. Later, it was rebranded under the name Web of Science Core Collection and was supplemented by many more citation indexes to maintain continual systematic updating of citation counts for journal articles [1]. The massive growth of medical and biological publications during this information era has led to new ways of assessing and systematically reviewing the impact of individual publications on a field of interest [2,3]. Thus, within the past two decades, there has been an influx of articles using citation index methods to evaluate the most relevant work within specific fields such as neurosurgery [1,4], plastic surgery [5], dermatology [6], orthopedic surgery [7], and others [8,9]. This review presents the 100 most highly cited journal articles in pediatric neurosurgery selected from eight journals using citation data accessed from the Web of Science Core Collection.

## Review

Methods

The focus of this study was to identify journal articles specifically dedicated to pediatric neurosurgery. Using similar methods as used by Ponce and Lozano, we identified eight journals by searching the Journal Citation Report database for publications with the words “pediatric neurosurgery” or “pediatric neuro” in the title [1,4]. The chosen journals were *Child’s Brain*, *Pediatric Neurosurgery*, *Child's Nervous System*, *Journal of Pediatrics*, *Journal of Neurosurgery*, *Journal of Neurosurgery-Pediatrics*, *Neuropediatrics*, and *Neurosurgery*. A search was then performed on the Web of Science Core Collection database by searching for each journal under “Publication Name” and using the Boolean “OR” function to separate fields. The results were ordered by the “Times Cited” category, which provided a list of all the articles from the eight journals appearing in the most cited order. The timeline used was from 1976 to 2021. The top 100 most cited articles were extracted from this list for analysis. The following variables were collected from each scientific article: publication journal, impact factor of journal, title, number of citations, year and month of publication, and type of article. Articles were excluded if they included basic science research, or animal studies, or were not purely pediatric or related to neurosurgery (i.e., they included adults or were not related to pediatric neurosurgery). The pediatric population was defined as ages 0-18 years as referenced in the articles. There were two exceptions to the age limit in the articles selected for review: in the study by Grant et al., the age limit was 21 (range 6 months to 21 years, with a mean of 10.4 ± 0.5 years), and in that by Tubbs et al., the age of patients ranged from 2 months to 20 years with a mean of 11 years [10,11].

Sources and citations

Eight journals were identified on the basis of the criteria described in Methods and the articles were sorted by most cited; 1609 pediatric neurosurgery journal articles were screened to select the 100 most cited articles since 1976 (Table 1).

**Table 1:** 2020 impact factors of selected journals

Name of journal	Impact factor
Child’s Brain	0.985
Child’s Nervous System	1.475
Journal of Neurosurgery	5.115
Journal of Neurosurgery-Pediatrics	2.375
Journal of Pediatrics	4.113
Neuropediatrics	1.947
Neurosurgery	3.968
Pediatric Neurosurgery	0.985

From these 100, 49 were published in *Journal of Neurosurgery*, 20 in *Journal of Pediatrics*, 17 in *Neurosurgery*, five in *Pediatric Neurosurgery*, four in *Child’s Nervous System*, two in *Neuropediatrics*, two in *Journal of Neurosurgery-Pediatrics*, and one in *Child’s Brain* (Table 2).

**Table 2:** The 100 most cited papers in pediatric neurosurgery

Rank	Citations	Article	Journal	Year of publication
1	4225	Papile LA, Burstein J, Burstein R, Koffler H. Incidence and evolution of subependymal and intraventricular hemorrhage: a study of infants with birth weights less than 1,500 gm. J Pediatr. 1978;92(4):529-534. doi: 10.1016/s0022-3476(78)80282-0	Journal of Pediatrics	1978
2	593	Lou HC, Lassen NA, Friis-Hansen B. Impaired autoregulation of cerebral blood flow in the distressed newborn infant. J Pediatr. 1979;94(1):118-121. doi: 10.1016/s0022-3476(79)80373-x	Journal of Pediatrics	1979
3	556	Daumas-Duport C, Scheithauer BW, Chodkiewicz JP, Laws ER Jr, Vedrenne C. Dysembryoplastic neuroepithelial tumor: a surgically curable tumor of young patients with intractable partial seizures. Report of thirty-nine cases. Neurosurgery. 1988;23(5):545-556. doi: 10.1227/00006123-198811000-00002	Neurosurgery	1988
4	555	Clarren SK, Alvord EC Jr, Sumi SM, Streissguth AP, Smith DW. Brain malformations related to prenatal exposure to ethanol. J Pediatr. 1978;92(1):64-67. doi: 10.1016/s0022-3476(78)80072-9	Journal of Pediatrics	1978
5	548	Rorke LB, Packer RJ, Biegel JA. Central nervous system atypical teratoid/rhabdoid tumors of infancy and childhood: definition of an entity. J Neurosurg. 1996;85(1):56-65. doi: 10.3171/jns.1996.85.1.0056	Journal of Neurosurgery	1996
6	478	Duhaime AC, Gennarelli TA, Thibault LE, Bruce DA, Margulies SS, Wiser R. The shaken baby syndrome. A clinical, pathological, and biomechanical study. J Neurosurg. 1987;66(3):409-415. doi: 10.3171/jns.1987.66.3.0409	Journal of Neurosurgery	1987
7	463	Drake JM, Kestle JR, Milner R, et al. Randomized trial of cerebrospinal fluid shunt valve design in pediatric hydrocephalus. Neurosurgery. 1998;43(2):294-305. doi: 10.1097/00006123-199808000-00068	Neurosurgery	1998
8	444	Papile LA, Munsick-Bruno G, Schaefer A. Relationship of cerebral intraventricular hemorrhage and early childhood neurologic handicaps. J Pediatr. 1983;103(2):273-277. doi: 10.1016/s0022-3476(83)80366-7	Journal of Pediatrics	1983
9	437	Evans AE, Jenkin RD, Sposto R, et al. The treatment of medulloblastoma. Results of a prospective randomized trial of radiation therapy with and without CCNU, vincristine, and prednisone. J Neurosurg. 1990;72(4):572-582. doi: 10.3171/jns.1990.72.4.0572	Journal of Neurosurgery	1990
10	436	Bowman RM, McLone DG, Grant JA, Tomita T, Ito JA. Spina bifida outcome: a 25-year prospective. Pediatr Neurosurg. 200;34(3):114-120. doi: 10.1159/000056005	Pediatric Neurosurgery	2001
11	391	Hoffman HJ, De Silva M, Humphreys RP, Drake JM, Smith ML, Blaser SI. Aggressive surgical management of craniopharyngiomas in children. J Neurosurg. 1992;76(1):47-52. doi: 10.3171/jns.1992.76.1.0047	Journal of Neurosurgery	1992
12	384	Renier D, Sainte-Rose C, Marchac D, Hirsch JF. Intracranial pressure in craniostenosis. J Neurosurg. 1982;57(3):370-377. doi: 10.3171/jns.1982.57.3.0370	Journal of Neurosurgery	1982
13	380	Packer RJ, Ater J, Allen J, et al. Carboplatin and vincristine chemotherapy for children with newly diagnosed progressive low-grade gliomas. J Neurosurg. 1997;86(5):747-754. doi: 10.3171/jns.1997.86.5.0747	Journal of Neurosurgery	1997
14	374	Bruce DA, Alavi A, Bilaniuk L, Dolinskas C, Obrist W, Uzzell B. Diffuse cerebral swelling following head injuries in children: the syndrome of "malignant brain edema". J Neurosurg. 1981;54(2):170-178. doi: 10.3171/jns.1981.54.2.0170	Journal of Neurosurgery	1981
15	360	Lovell MR, Collins MW, Iverson GL, et al. Recovery from mild concussion in high school athletes. J Neurosurg. 2003;98(2):296-301. doi: 10.3171/jns.2003.98.2.0296	Journal of Neurosurgery	2003
16	358	Inder TE, Wells SJ, Mogridge NB, Spencer C, Volpe JJ. Defining the nature of the cerebral abnormalities in the premature infant: a qualitative magnetic resonance imaging study. J Pediatr. 2003;143(2):171-179. doi: 10.1067/S0022-3476(03)00357-3	Journal of Pediatrics	2003
17	350	Packer RJ, Sutton LN, Elterman R, et al. Outcome for children with medulloblastoma treated with radiation and cisplatin, CCNU, and vincristine chemotherapy. J Neurosurg. 1994;81(5):690-698. doi: 10.3171/jns.1994.81.5.0690	Journal of Neurosurgery	1994
18	350	Scott RM, Smith JL, Robertson RL, Madsen JR, Soriano SG, Rockoff MA. Long-term outcome in children with moyamoya syndrome after cranial revascularization by pial synangiosis. J Neurosurg. 2004;100(2 Suppl Pediatrics):142-149. doi: 10.3171/ped.2004.100.2.0142	Journal of Neurosurgery	2004
19	345	Listernick R, Charrow J, Greenwald M, Mets M. Natural history of optic pathway tumors in children with neurofibromatosis type 1: a longitudinal study. J Pediatr. 1994;125(1):63-66. doi: 10.1016/s0022-3476(94)70122-9	Journal of Pediatrics	1994
20	344	Pang D, Wilberger JE Jr. Spinal cord injury without radiographic abnormalities in children. J Neurosurg. 1982;57(1):114-129. doi: 10.3171/jns.1982.57.1.0114	Journal of Neurosurgery	1982
21	330	Berger MS, Kincaid J, Ojemann GA, Lettich E. Brain mapping techniques to maximize resection, safety, and seizure control in children with brain tumors. Neurosurgery. 1989;25(5):786-792. doi: 10.1097/00006123-198911000-00015	Neurosurgery	1989
22	327	Chasnoff IJ, Bussey ME, Savich R, Stack CM. Perinatal cerebral infarction and maternal cocaine use. J Pediatr. 1986;108(3):456-459. doi: 10.1016/s0022-3476(86)80896-4	Journal of Pediatrics	1986
23	329	Miller SP, Ramaswamy V, Michelson D, et al. Patterns of brain injury in term neonatal encephalopathy. J Pediatr. 2005;146(4):453-460. doi: 10.1016/j.jpeds.2004.12.026	Journal of Pediatrics	2005
24	329	Taylor A, Butt W, Rosenfeld J, et al. A randomized trial of very early decompressive craniectomy in children with traumatic brain injury and sustained intracranial hypertension. Childs Nerv Syst. 2001;17(3):154-162. doi: 10.1007/s003810000410	Child’s Nervous System	2001
25	328	Barkovich AJ, Kuzniecky RI, Dobyns WB, Jackson GD, Becker LE, Evrard P. A classification scheme for malformations of cortical development. Neuropediatrics. 1996;27(2):59-63. doi: 10.1055/s-2007-973750	Neuropediatrics	1996
26	313	Miller SP, Ferriero DM, Leonard C, et al. Early brain injury in premature newborns detected with magnetic resonance imaging is associated with adverse early neurodevelopmental outcome. J Pediatr. 2005;147(5):609-616. doi: 10.1016/j.jpeds.2005.06.033	Journal of Pediatrics	2005
27	302	Collins MW, Lovell MR, Iverson GL, Cantu RC, Maroon JC, Field M. Cumulative effects of concussion in high school athletes. Neurosurgery. 2002;51(5):1175-1181. doi: 10.1097/00006123-200211000-00011	Neurosurgery	2002
28	301	Sainte-Rose C, Piatt JH, Renier D, et al. Mechanical complications in shunts. Pediatr Neurosurg. 1991;17(1):2-9. doi: 10.1159/000120557	Pediatric Neurosurgery	1992
29	296	Bruce DA, Schut L, Bruno LA, Wood JH, Sutton LN. Outcome following severe head injuries in children. J Neurosurg. 1978;48(5):679-688. doi: 10.3171/jns.1978.48.5.0679	Journal of Neurosurgery	1978
30	286	Jones RF, Stening WA, Brydon M. Endoscopic third ventriculostomy. Neurosurgery. 1990;26(1):86-92. doi: 10.1097/00006123-199001000-00012	Neurosurgery	1990
31	285	Balkaran B, Char G, Morris JS, Thomas PW, Serjeant BE, Serjeant GR. Stroke in a cohort of patients with homozygous sickle cell disease. J Pediatr. 1992;120(3):360-366. doi: 10.1016/s0022-3476(05)80897-2	Journal of Pediatrics	1992
32	282	Barlow CF, Priebe CJ, Mulliken JB, et al. Spastic diplegia as a complication of interferon alfa-2a treatment of hemangiomas of infancy. J Pediatr. 1998;132(3 Pt 1):527-530. doi: 10.1016/s0022-3476(98)70034-4	Journal of Pediatrics	1998
33	280	Hoffman HJ, Otsubo H, Hendrick EB, et al. Intracranial germ-cell tumors in children. J Neurosurg. 1991;74(4):545-551. doi: 10.3171/jns.1991.74.4.0545	Journal of Neurosurgery	1991
34	276	Rickert CH, Paulus W. Epidemiology of central nervous system tumors in childhood and adolescence based on the new WHO classification. Childs Nerv Syst. 2001;17(9):503-511. doi: 10.1007/s003810100496	Child’s Nervous System	2001
35	267	Hoffman HJ, Hendrick EB, Humphreys RP. The tethered spinal cord: its protean manifestations, diagnosis and surgical correction. Childs Brain. 1976;2(3):145-155. doi: 10.1159/000119610	Child’s Brain	1976
36	265	Kestle J, Drake J, Milner R, et al. Long-term follow-up data from the Shunt Design Trial. Pediatr Neurosurg. 2000;33(5):230-236. doi: 10.1159/000055960	Pediatric Neurosurgery	2000
37	265	Choux M, Genitori L, Lang D, Lena G. Shunt implantation: reducing the incidence of shunt infection. J Neurosurg. 1992;77(6):875-880. doi: 10.3171/jns.1992.77.6.0875	Journal of Neurosurgery	1992
38	261	Packer RJ, Sutton LN, Atkins TE, et al. A prospective study of cognitive function in children receiving whole-brain radiotherapy and chemotherapy: 2-year results. J Neurosurg. 1989;70(5):707-713. doi: 10.3171/jns.1989.70.5.0707	Journal of Neurosurgery	1989
39	255	Pollack IF, Polinko P, Albright AL, Towbin R, Fitz C. Mutism and pseudobulbar symptoms after resection of posterior fossa tumors in children: incidence and pathophysiology. Neurosurgery. 1995;37(5):885-893. doi: 10.1227/00006123-199511000-00006	Neurosurgery	1995
40	254	Albright AL, Packer RJ, Zimmerman R, Rorke LB, Boyett J, Hammond GD. Magnetic resonance scans should replace biopsies for the diagnosis of diffuse brain stem gliomas: a report from the Children's Cancer Group. Neurosurgery. 1993;33(6):1026-1030. doi: 10.1227/00006123-199312000-00010	Neurosurgery	1993
41	252	Park TS, Hoffman HJ, Hendrick EB, Humphreys RP, Becker LE. Medulloblastoma: clinical presentation and management. Experience at the hospital for sick children, Toronto, 1950-1980. J Neurosurg. 1983;58(4):543-552. doi: 10.3171/jns.1983.58.4.0543	Journal of Neurosurgery	1983
42	251	Pegelow CH, Adams RJ, McKie V, et al. Risk of recurrent stroke in patients with sickle cell disease treated with erythrocyte transfusions. J Pediatr. 1995;126(6):896-899. doi: 10.1016/s0022-3476(95)70204-0	Journal of Pediatrics	1995
43	250	Maalouf EF, Duggan PJ, Rutherford MA, et al. Magnetic resonance imaging of the brain in a cohort of extremely preterm infants. J Pediatr. 1999;135(3):351-357. doi: 10.1016/s0022-3476(99)70133-2	Journal of Pediatrics	1999
44	247	Puget S, Garnett M, Wray A, et al. Pediatric craniopharyngiomas: classification and treatment according to the degree of hypothalamic involvement. J Neurosurg. 2007;106(1 Suppl):3-12. doi: 10.3171/ped.2007.106.1.3	Journal of Neurosurgery	2007
45	244	Matsushima T, Inoue T, Suzuki SO, Fujii K, Fukui M, Hasuo K. Surgical treatment of moyamoya disease in pediatric patients--comparison between the results of indirect and direct revascularization procedures. Neurosurgery. 1992;31(3):401-405. doi: 10.1227/00006123-199209000-00003	Neurosurgery	1992
46	238	Robertson PL, Zeltzer PM, Boyett JM, et al. Survival and prognostic factors following radiation therapy and chemotherapy for ependymomas in children: a report of the Children's Cancer Group. J Neurosurg. 1998;88(4):695-703. doi: 10.3171/jns.1998.88.4.0695	Journal of Neurosurgery	1998
47	237	Glass HC, Glidden D, Jeremy RJ, Barkovich AJ, Ferriero DM, Miller SP. Clinical neonatal seizures are independently associated with outcome in infants at risk for hypoxic-ischemic brain injury. J Pediatr. 2009;155(3):318-323. doi: 10.1016/j.jpeds.2009.03.040	Journal of Pediatrics	2009
48	240	Kulkarni AV, Drake JM, Lamberti-Pasculli M. Cerebrospinal fluid shunt infection: a prospective study of risk factors. J Neurosurg. 2001;94(2):195-201. doi: 10.3171/jns.2001.94.2.0195	Journal of Neurosurgery	2001
49	235	Cinalli G, Sainte-Rose C, Chumas P, et al. Failure of third ventriculostomy in the treatment of aqueductal stenosis in children. J Neurosurg. 1999;90(3):448-454. doi: 10.3171/jns.1999.90.3.0448	Journal of Neurosurgery	1999
50	235	Ellenberg L, McComb JG, Siegel SE, Stowe S. Factors affecting intellectual outcome in pediatric brain tumor patients. Neurosurgery. 1987;21(5):638-644. doi: 10.1227/00006123-198711000-00006	Neurosurgery	1987
51	233	Pollack IF, Gerszten PC, Martinez AJ, et al. Intracranial ependymomas of childhood: long-term outcome and prognostic factors. Neurosurgery. 1995;37(4):655-667. doi: 10.1227/00006123-199510000-00008	Neurosurgery	1995
52	229	Bada HS, Hajjar W, Chua C, Sumner DS. Noninvasive diagnosis of neonatal asphyxia and intraventricular hemorrhage by Doppler ultrasound. J Pediatr. 1979;95(5 Pt 1):775-779. doi: 10.1016/s0022-3476(79)80735-0	Journal of Pediatrics	1979
53	229	De Vile CJ, Grant DB, Kendall BE, et al. Management of childhood craniopharyngioma: can the morbidity of radical surgery be predicted? J Neurosurg. 1996;85(1):73-81. doi: 10.3171/jns.1996.85.1.0073	Journal of Neurosurgery	1996
54	226	Hadley MN, Zabramski JM, Browner CM, Rekate H, Sonntag VK. Pediatric spinal trauma. Review of 122 cases of spinal cord and vertebral column injuries. J Neurosurg. 1988;68(1):18-24. doi: 10.3171/jns.1988.68.1.0018	Journal of Neurosurgery	1988
55	223	Renier D, Lajeunie E, Arnaud E, Marchac D. Management of craniosynostoses. Childs Nerv Syst. 2000;16(10-11):645-658. doi: 10.1007/s003810000320	Child’s Nervous System	2000
56	225	Tuli S, Drake J, Lawless J, Wigg M, Lamberti-Pasculli M. Risk factors for repeated cerebrospinal shunt failures in pediatric patients with hydrocephalus. J Neurosurg. 2000;92(1):31-38. doi: 10.3171/jns.2000.92.1.0031	Journal of Neurosurgery	2000
57	221	Epstein F, McCleary EL. Intrinsic brain-stem tumors of childhood: surgical indications. J Neurosurg. 1986;64(1):11-15. doi: 10.3171/jns.1986.64.1.0011	Journal of Neurosurgery	1986
58	220	Grant GA, Jolley M, Ellenbogen RG, Roberts TS, Gruss JR, Loeser JD. Failure of autologous bone-assisted cranioplasty following decompressive craniectomy in children and adolescents. J Neurosurg. 2004;100(2 Suppl Pediatrics):163-168. doi: 10.3171/ped.2004.100.2.0163	Journal of Neurosurgery	2004
59	218	Adelson PD, Ragheb J, Kanev P, et al. Phase II clinical trial of moderate hypothermia after severe traumatic brain injury in children. Neurosurgery. 2005;56(4):740-754. doi: 10.1227/01.neu.0000156471.50726.26	Neurosurgery	2005
60	213	Tubbs RS, McGirt MJ, Oakes WJ. Surgical experience in 130 pediatric patients with Chiari I malformations. J Neurosurg. 2003;99(2):291-296. doi: 10.3171/jns.2003.99.2.0291	Journal of Neurosurgery	2003
61	210	Constantini S, Miller DC, Allen JC, Rorke LB, Freed D, Epstein FJ. Radical excision of intramedullary spinal cord tumors: surgical morbidity and long-term follow-up evaluation in 164 children and young adults. J Neurosurg. 2000;93(2 Suppl):183-193. doi: 10.3171/spi.2000.93.2.0183	Journal of Neurosurgery	2000
62	206	Moser RS, Schatz P, Jordan BD. Prolonged effects of concussion in high school athletes. Neurosurgery. 2005;57(2):300-306. doi: 10.1227/01.neu.0000166663.98616.e4	Neurosurgery	2005
63	205	Nazar GB, Hoffman HJ, Becker LE, Jenkin D, Humphreys RP, Hendrick EB. Infratentorial ependymomas in childhood: prognostic factors and treatment. J Neurosurg. 1990;72(3):408-417. doi: 10.3171/jns.1990.72.3.0408	Journal of Neurosurgery	1990
64	204	VandenBerg SR, May EE, Rubinstein LJ, et al. Desmoplastic supratentorial neuroepithelial tumors of infancy with divergent differentiation potential ("desmoplastic infantile gangliogliomas"). Report on 11 cases of a distinctive embryonal tumor with favorable prognosis. J Neurosurg. 1987;66(1):58-71. doi: 10.3171/jns.1987.66.1.0058	Journal of Neurosurgery	1987
65	203	Albright AL, Wisoff JH, Zeltzer PM, Boyett JM, Rorke LB, Stanley P. Effects of medulloblastoma resections on outcome in children: a report from the Children's Cancer Group. Neurosurgery. 1996;38(2):265-271. doi: 10.1097/00006123-199602000-00007	Neurosurgery	1996
66	206	O'Hayon BB, Drake JM, Ossip MG, Tuli S, Clarke M. Frontal and occipital horn ratio: A linear estimate of ventricular size for multiple imaging modalities in pediatric hydrocephalus. Pediatr Neurosurg. 1998;29(5):245-249. doi: 10.1159/000028730	Pediatric Neurosurgery	1998
67	204	Pierre-Kahn A, Zerah M, Renier D, et al. Congenital lumbosacral lipomas. Childs Nerv Syst. 1997;13(6):298-335. doi: 10.1007/s003810050090	Child’s Nervous System	1997
68	204	Edwards MS, Hudgins RJ, Wilson CB, Levin VA, Wara WM. Pineal region tumors in children. J Neurosurg. 1988;68(5):689-697. doi: 10.3171/jns.1988.68.5.0689	Journal of Neurosurgery	1988
69	199	Robertson PL, Muraszko KM, Holmes EJ, et al. Incidence and severity of postoperative cerebellar mutism syndrome in children with medulloblastoma: a prospective study by the Children's Oncology Group. J Neurosurg. 2006;105(6 Suppl):444-451. doi: 10.3171/ped.2006.105.6.444	Journal of Neurosurgery	2006
70	198	Hirsch JF, Pierre-Kahn A, Renier D, Sainte-Rose C, Hoppe-Hirsch E. The Dandy-Walker malformation. A review of 40 cases. J Neurosurg. 1984;61(3):515-522. doi: 10.3171/jns.1984.61.3.0515	Journal of Neurosurgery	1984
71	194	Wisoff JH, Boyett JM, Berger MS, et al. Current neurosurgical management and the impact of the extent of resection in the treatment of malignant gliomas of childhood: a report of the Children's Cancer Group trial no. CCG-945. J Neurosurg. 1998;89(1):52-59. doi: 10.3171/jns.1998.89.1.0052	Journal of Neurosurgery	1998
72	194	Teo C, Jones R. Management of hydrocephalus by endoscopic third ventriculostomy in patients with myelomeningocele. Pediatr Neurosurg. 1996;25(2):57-63. doi: 10.1159/000121098	Pediatric Neurosurgery	1996
73	194	Perlman JM, Hill A, Volpe JJ. The effect of patent ductus arteriosus on flow velocity in the anterior cerebral arteries: ductal steal in the premature newborn infant. J Pediatr. 1981;99(5):767-771. doi: 10.1016/s0022-3476(81)80408-8	Journal of Pediatrics	1981
74	194	Hoffman HJ, Taecholarn C, Hendrick EB, Humphreys RP. Management of lipomyelomeningoceles. Experience at the Hospital for Sick Children, Toronto. J Neurosurg. 1985;62(1):1-8. doi: 10.3171/jns.1985.62.1.0001	Journal of Neurosurgery	1985
75	193	Bada HS, Korones SB, Perry EH, et al. Mean arterial blood pressure changes in premature infants and those at risk for intraventricular hemorrhage. J Pediatr. 1990;117(4):607-614. doi: 10.1016/s0022-3476(05)80700-0	Journal of Pediatrics	1990
76	192	Keucher TR, Mealey J Jr. Long-term results after ventriculoatrial and ventriculoperitoneal shunting for infantile hydrocephalus. J Neurosurg. 1979;50(2):179-186. doi: 10.3171/jns.1979.50.2.0179	Journal of Neurosurgery	1979
77	191	Harsh GR 4th, Edwards MS, Wilson CB. Intracranial arachnoid cysts in children. J Neurosurg. 1986;64(6):835-842. doi: 10.3171/jns.1986.64.6.0835	Journal of Neurosurgery	1986
78	189	Warf BC. Comparison of endoscopic third ventriculostomy alone and combined with choroid plexus cauterization in infants younger than 1 year of age: a prospective study in 550 African children. J Neurosurg. 2005;103(6 Suppl):475-481. doi: 10.3171/ped.2005.103.6.0475	Journal of Neurosurgery	2005
79	187	Jimenez DF, Barone CM. Endoscopic craniectomy for early surgical correction of sagittal craniosynostosis. J Neurosurg. 1998;88(1):77-81. doi: 10.3171/jns.1998.88.1.0077	Journal of Neurosurgery	1998
80	187	Muizelaar JP, Marmarou A, DeSalles AA, et al. Cerebral blood flow and metabolism in severely head-injured children. Part 1: Relationship with GCS score, outcome, ICP, and PVI. J Neurosurg. 1989;71(1):63-71. doi: 10.3171/jns.1989.71.1.0063	Journal of Neurosurgery	1989
81	184	Ciricillo SF, Cogen PH, Harsh GR, Edwards MS. Intracranial arachnoid cysts in children. A comparison of the effects of fenestration and shunting. J Neurosurg. 1991;74(2):230-235. doi:10.3171/jns.1991.74.2.0230	Journal of Neurosurgery	1991
82	183	Levin HS, Aldrich EF, Saydjari C, et al. Severe head injury in children: experience of the Traumatic Coma Data Bank. Neurosurgery. 1992;31(3):435-444. doi: 10.1227/00006123-199209000-00008	Neurosurgery	1992
83	183	Shankaran S, Slovis TL, Bedard MP, Poland RL. Sonographic classification of intracranial hemorrhage. A prognostic indicator of mortality, morbidity, and short-term neurologic outcome. J Pediatr. 1982;100(3):469-475. doi: 10.1016/s0022-3476(82)80462-9	Journal of Pediatrics	1982
84	182	Hadley MN, Sonntag VK, Rekate HL, Murphy A. The infant whiplash-shake injury syndrome: a clinical and pathological study. Neurosurgery. 1989;24(4):536-540. doi: 10.1227/00006123-198904000-00008	Neurosurgery	1989
85	181	Ruge JR, Sinson GP, McLone DG, Cerullo LJ. Pediatric spinal injury: the very young. J Neurosurg. 1988;68(1):25-30. doi: 10.3171/jns.1988.68.1.0025	Journal of Neurosurgery	1988
86	180	Gilbert JN, Jones KL, Rorke LB, Chernoff GF, James HE. Central nervous system anomalies associated with meningomyelocele, hydrocephalus, and the Arnold-Chiari malformation: reappraisal of theories regarding the pathogenesis of posterior neural tube closure defects. Neurosurgery. 1986;18(5):559-564. doi: 10.1227/00006123-198605000-00008	Neurosurgery	1986
87	183	Tubbs RS, Beckman J, Naftel RP, et al. Institutional experience with 500 cases of surgically treated pediatric Chiari malformation Type I. J Neurosurg Pediatr. 2011;7(3):248-256. doi: 10.3171/2010.12.PEDS10379	Journal of Neurosurgery-Pediatrics	2011
88	181	Berger RP, Adelson PD, Pierce MC, Dulani T, Cassidy LD, Kochanek PM. Serum neuron-specific enolase, S100B, and myelin basic protein concentrations after inflicted and noninflicted traumatic brain injury in children. J Neurosurg. 2005;103(1 Suppl):61-68. doi: 10.3171/ped.2005.103.1.0061	Journal of Neurosurgery	2005
89	179	Karasawa J, Touho H, Ohnishi H, Miyamoto S, Kikuchi H. Long-term follow-up study after extracranial-intracranial bypass surgery for anterior circulation ischemia in childhood moyamoya disease. J Neurosurg. 1992;77(1):84-89. doi: 10.3171/jns.1992.77.1.0084	Journal of Neurosurgery	1992
90	179	Ginsburg HH, Shetter AG, Raudzens PA. Postoperative paraplegia with preserved intraoperative somatosensory evoked potentials. Case report. J Neurosurg. 1985;63(2):296-300. doi: 10.3171/jns.1985.63.2.0296	Journal of Neurosurgery	1985
91	178	Collins M, Lovell MR, Iverson GL, Ide T, Maroon J. Examining concussion rates and return to play in high school football players wearing newer helmet technology: a three-year prospective cohort study. Neurosurgery. 2006;58(2):275-286. doi: 10.1227/01.NEU.0000200441.92742.46	Neurosurgery	2006
92	177	Simon TD, Riva-Cambrin J, Srivastava R, et al. Hospital care for children with hydrocephalus in the United States: utilization, charges, comorbidities, and deaths. J Neurosurg Pediatr. 2008;1(2):131-137. doi: 10.3171/PED/2008/1/2/131	Journal of Neurosurgery-Pediatrics	2008
93	177	Allen JC, Kim JH, Packer RJ. Neoadjuvant chemotherapy for newly diagnosed germ-cell tumors of the central nervous system. J Neurosurg. 1987;67(1):65-70. doi: 10.3171/jns.1987.67.1.0065	Journal of Neurosurgery	1987
94	177	Thomsett MJ, Conte FA, Kaplan SL, Grumbach MM. Endocrine and neurologic outcome in childhood craniopharyngioma: review of effect of treatment in 42 patients. J Pediatr. 1980;97(5):728-735. doi: 10.1016/s0022-3476(80)80254-x	Journal of Pediatrics	1980
95	176	Warf BC. Hydrocephalus in Uganda: the predominance of infectious origin and primary management with endoscopic third ventriculostomy. J Neurosurg. 2005;102(1 Suppl):1-15. doi: 10.3171/ped.2005.102.1.0001	Journal of Neurosurgery	2005
96	176	Pollack IF, Claassen D, al-Shboul Q, Janosky JE, Deutsch M. Low-grade gliomas of the cerebral hemispheres in children: an analysis of 71 cases. J Neurosurg. 1995;82(4):536-547. doi: 10.3171/jns.1995.82.4.0536	Journal of Neurosurgery	1995
97	175	Albright AL, Guthkelch AN, Packer RJ, Price RA, Rourke LB. Prognostic factors in pediatric brain-stem gliomas. J Neurosurg. 1986;65(6):751-755. doi: 10.3171/jns.1986.65.6.0751	Journal of Neurosurgery	1986
98	174	Kulkarni AV, Drake JM, Mallucci CL, et al. Endoscopic third ventriculostomy in the treatment of childhood hydrocephalus. J Pediatr. 2009;155(2):254-9.e1. doi: 10.1016/j.jpeds.2009.02.048	Journal of Pediatrics	2009
99	174	Sekhar LN, Moossy J, Guthkelch AN. Malfunctioning ventriculoperitoneal shunts. Clinical and pathological features. J Neurosurg. 1982;56(3):411-416. doi: 10.3171/jns.1982.56.3.0411	Journal of Neurosurgery	1982
100	173	Hoffmann GF, Athanassopoulos S, Burlina AB, et al. Clinical course, early diagnosis, treatment, and prevention of disease in glutaryl-CoA dehydrogenase deficiency. Neuropediatrics. 1996;27(3):115-123. doi: 10.1055/s-2007-973761	Neuropediatrics	1996

Field of study

The categorization of journal articles was similar to that by Ponce and Lozano and Khan et al.: congenital, functional, hydrocephalus, spine, tumor, trauma, vascular, and other (Table 3) [1,12].

**Table 3:** Type and field of study

Study type	Congenital	Functional	Hydrocephalus	Spine	Tumor	Trauma	Vascular	Other	Total
Case report	1	1	0	0	2	1	3	0	8
Comparative	1	0	4	0	1	4	1	0	11
Clinical trial	0	0	2	0	7	2	1	0	12
Cross-sectional	0	0	1	0	0	0	0	0	1
Multi-center	0	0	1	0	0	0	0	0	1
Original	3	4	5	1	11	4	5	1	34
Review	4	1	3	3	12	3	3	4	33
Total	9	6	16	4	33	14	13	5	100

The field of study with most publications in pediatric neurosurgery was tumor-related (n=33), followed by hydrocephalus (n=16), trauma (n=14), congenital (n=9), functional (n=6), vascular (n=5), spinal (n=4), and other (n=5). Gliomas were the most commonly discussed tumors in the pediatric population, contributing to six of the 33 total journal articles. There were four articles related to each tumor type: craniopharyngioma, medulloblastomas, and ependymomas. Other tumor-related articles were of origins such as spine and neuroepithelial. Hydrocephalus was the second most discussed topic, with findings related to shunt design, malfunctions, and improvements. Among the 14 articles describing trauma in the pediatric population, severe traumatic brain injury (n=5) and concussion (n=4) were the most common topics. The reviewed articles from the vascular field were related to abnormal cerebral blood flow, intraventricular hemorrhage, and revascularization. Topics that did not fit the listed categories were listed as “Other” and included articles related to magnetic resonance imaging (MRI) findings for non-traumatic brain injury, craniectomy, and neurological problems associated with preterm infants.

Type of study

The articles were also categorized by time of study, based on the information from the article or the category assigned by PubMed (Table 3). The 100 selected journal articles comprised 34 original articles, 33 reviews, 12 clinical trials, 11 comparative studies, and 8 case report/reviews; there was also one cross-sectional and one multi-center study. Over two-thirds of the most cited pediatric neurosurgical articles were either original (prospective, non-review) or review (retrospective with regard to data collection and analysis). Clinical trials were most common in articles related to tumors (n=7), hydrocephalus (n=2), trauma (n=2), and vascular (n=1). Although hydrocephalus and trauma were the second and third most commonly studied fields, they each had four comparative studies, the rest of the comparative studies being congenital (n=1), trauma (n=1), and vascular (n=1). Case reports were most common in the vascular field (n=3), followed by tumor-related (n=2), trauma (n=1), functional (n=1), and congenital (n=1). Both the cross-sectional and multi-center studies were related to hydrocephalus.

Discussion

In this literature review, we present the top 100 most cited articles in pediatric neurosurgery since 1976. Our research was conducted similarly to other reviews of the most relevant articles within their respective fields [1,4-9]. To keep this article focused solely on clinical neurosurgery in the pediatric population, we excluded basic science research, animal studies, and any articles that included an adult population. This article contributes significantly to neurosurgery, as pediatric neurosurgery is a specific and more recent subspecialty that requires its own analysis. Although Ponce and Lozano [1,4] published two articles on the most cited work in neurosurgery, only three of their 100 are consistent with pediatric neurosurgical findings, regarding neuroepithelial tumors and the positive effects of surgical resection [13], randomized trial for treatment of medulloblastoma [14], and findings concerning shaken baby syndrome [15]. Furthermore, in 2013, Wilcox et al. compiled and categorized the most cited work in pediatric neurosurgery; however, they only evaluated four clinical pediatric neurosurgical journals and included basic science research within the criteria for relevance [16]. This work was continued by Khan et al. in 2013, in Part 2, with a focus on non-pediatric journals only, excluding basic science, imaging, histology, pathology and pharmacology-related articles from their selection of the top 100 [12].

Overall, the present study contributes to the field of pediatric neurosurgery by compiling pediatric neurosurgical articles in a single review, with a specific focus on pediatric clinical neurosurgical studies from pediatric and non-pediatric surgical journals and non-surgical journals. Furthermore, by grouping the 100 most cited pediatric neurosurgical articles in the field within one table, we hope to eliminate the time burden for other physicians, residents, and others interested in learning about the pediatric neurosurgery field.

Limitations

Several limitations are associated with the citation analysis and the impact of journal articles. There is still a debate about correlating the number of citations with the importance of an article [3]. The journals selected do not encompass all those in which highly cited articles in pediatric neurosurgery can be published, so seminal articles could have been missed. Owing to the time frame of the literature search, more recent articles that could have a high impact might not yet have had time to accumulate the citations necessary to be included in the rankings [17]. The limitations of the Web of Science database must also be considered as it only includes citation data from 1976 to 2021, so older journal articles, or very new ones, will not necessarily be well represented. Other databases such as Google Scholar (2,689,809) or Microsoft Academic (1,840,702) also contain more citations than the Web of Science Core Collection (1,503,657) [2].

## Conclusions

Using the Web of Science Core Collection database, the top 100 articles in pediatric neurosurgery from the selected journals were identified and citation analysis was used to identify the most impactful articles. The compilation could help clinicians and researchers to familiarize themselves with the journal articles included in terms of study type, study field, journal of publication, and recurring authors. Although there might not be a direct impact of such an article on clinical practice, a review of this nature is of archival value and shows pediatric neurosurgeons in the field what papers are of most value to others who have cited their works.
